# Exploring Codon Optimization and Response Surface Methodology to Express Biologically Active Transmembrane RANKL in *E. coli*

**DOI:** 10.1371/journal.pone.0096259

**Published:** 2014-05-08

**Authors:** Sushila Maharjan, Bijay Singh, Jin-Duck Bok, Jeong-In Kim, Tao Jiang, Chong-Su Cho, Sang-Kee Kang, Yun-Jaie Choi

**Affiliations:** 1 Department of Agricultural Biotechnology, Seoul National University, Seoul, Republic of Korea; 2 Research Institute for Agriculture and Life Sciences, Seoul National University, Seoul, Republic of Korea; 3 Research Institute of Eco-friendly Animal Science, Institute of Green-Bio Science and Technology, Seoul National University, Kangwon-Do, Republic of Korea; National University of Singapore, Singapore

## Abstract

Receptor activator of nuclear factor (NF)-κB ligand (RANKL), a master cytokine that drives osteoclast differentiation, activation and survival, exists in both transmembrane and extracellular forms. To date, studies on physiological role of RANKL have been mainly carried out with extracellular RANKL probably due to difficulties in achieving high level expression of functional transmembrane RANKL (mRANKL). In the present study, we took advantage of codon optimization and response surface methodology to optimize the soluble expression of mRANKL in *E. coli*. We optimized the codon usage of mRANKL sequence to a preferred set of codons for *E. coli* changing its codon adaptation index from 0.64 to 0.76, tending to increase its expression level in *E. coli*. Further, we utilized central composite design to predict the optimum combination of variables (cell density before induction, lactose concentration, post-induction temperature and post-induction time) for the expression of mRANKL. Finally, we investigated the effects of various experimental parameters using response surface methodology. The best combination of response variables was 0.6 OD_600_, 7.5 mM lactose, 26°C post-induction temperature and 5 h post-induction time that produced 52.4 mg/L of fusion mRANKL. Prior to functional analysis of the protein, we purified mRANKL to homogeneity and confirmed the existence of trimeric form of mRANKL by native gel electrophoresis and gel filtration chromatography. Further, the biological activity of mRANKL to induce osteoclast formation on RAW264.7 cells was confirmed by tartrate resistant acid phosphatase assay and quantitative real-time polymerase chain reaction assays. Importantly, a new finding from this study was that the biological activity of mRANKL is higher than its extracellular counterpart. To the best of our knowledge, this is the first time to report heterologous expression of mRANKL in soluble form and to perform a comparative study of functional properties of both forms of RANKL.

## Introduction

Receptor activator of nuclear factor (NF)-κB ligand (RANKL), a member of tumor necrosis factor (TNF) superfamily, is preferentially expressed on osteoblast and stromal lineage cells whereas its receptor RANK is preferentially expressed on osteoclast lineage cells [1]–[3]. RANKL is produced as a type II transmembrane protein on these cells, and cleaved into an extracellular soluble form by specific metalloproteinases [4]–[7]. The latter form has high similarity to TNF-related apoptosis inducing ligand (TRAIL), FasL (TNF-related ligand) and TNF itself [8]. RANKL and RANK are best known for their essential role in controlling osteoclastogenesis. The essential physiological roles of RANKL-RANK have been elucidated through several in vitro and in vivo studies. It is now known that RANKL-RANK system is required to trigger a network of intracellular signaling cascades that promote osteoclast differentiation, activation and survival [9]. Moreover, RANKL is essential to induce expression of genes including tartrate-resistant acid phosphatase (*Trap*), calcitonin receptor (*CalcR*), cathepsin K (*CtsK*), receptor for macrophage-colony-stimulating factor (*Cfms*) and nuclear factor of activated T-cells (*Nfatc1*) leading to the development of mature osteoclasts [9]–[11]. The biological activity of RANKL is balanced by its physiological decoy receptor, osteoprotegerin (OPG) that competes with RANK for RANKL and thus dictates the quantity of bone resorbed [12]–[14]. The elucidation of the signaling pathway mediated by OPG, RANK and RANKL provided a major breakthrough that clarified the role played by RANKL in osteoclast biology [15].

RANKL-RANK signaling is not only related to bone physiology but also has essential roles in maturation and activation of the immune system. RANKL-RANK system is also functionally involved in lymph node organogenesis, development of thymic medullary epithelial cells [16]–[20], central thermoregulation [21], development of a lactating mammary gland during pregnancy [22], promotes dendritic cell survival [2] and normal development of Peyer's patches. Recently, Knoop et al. showed that RANKL is the critical factor that controls the differentiation of M cells from RANK-expressing intestinal epithelial precursor cells. As a consequence, the RANKL null mice failed to develop M cells in their Peyer's patches which could be restored by systemic administration of exogenous RANKL [23].

Despite the diverse roles of RANKL in various cells, the physiological roles of RANKL are mainly studied using extracellular form due to its soluble nature. So, it is necessary to conduct experimental studies in order to shed light on the function of transmembrane RANKL. But, large quantities of functional proteins are required for biophysical characterization and other studies. Because the use of mammalian expression systems is usually expensive, time-consuming and difficult to scale up while providing optimal conditions for the expression of mammalian proteins themselves, *E. coli* can be used as a feasible alternative host to eukaryotic cells for the overexpression of mammalian proteins. The use of *E. coli* expression systems for heterologous production of protein by recombinant DNA technology has long been established. *E. coli* overexpression system provides the advantages of inexpensiveness, fast growth, straight forward genetics, a large number of mutant host strains and expression vectors, scalability and high expression [24]–[26].

However, bacteria and mammals prefer to use different codons and thus, biased codon usage is one of the major factors affecting the heterologous expression of mammalian protein in *E. coli* [27]. The problem of codon bias can be resolved by codon optimization, a genetic technique which involves the replacement of existing rare codons of a species with a set of more favorable host codons throughout the whole gene to achieve optimum expression of a foreign gene in a host's cellular system [27]–[29]. Accordingly, synthesis of the target gene is often faster and cheaper to get the codon-optimized gene. Gene synthesis offers the additional benefit that most gene optimization algorithms optimize not only rare codons but also mRNA secondary structure, the latter affects the translation efficiency [30].

Considerably, protein expression is influenced by multiple parameters including selection of vector with an appropriate promoter, fusion tag, expression host strain and the expression conditions such as temperature, concentration of inducer, induction time and composition of the culture medium. These parameters can be optimized to improve the yield of expressed proteins [31]. Optimization of protein expression and production can be achieved by a conventional one-factor-at-a-time approach [32]. In this method, optimization is usually done by varying a single factor, while keeping all the other factors fixed at a given set of conditions. But, this method is not only time consuming, but also incapable of achieving the true optimal conditions because the method ignores the interactions between the influencing factors [33]. Alternatively, response surface methodology (RSM) can be used to determine the individual role of each factor as well as their influences among the factors. RSM is a mathematical and statistical tool for designing experiments, building models, evaluating the effects of several factors, and achieving the optimum conditions for desirable responses with a limited number of experiments [34].

In this study, we utilized codon optimization strategy and response surface methodology to assess the heterologous production of both transmembrane and extracellular forms of RANKL as soluble proteins in *E. coli*. We optimized and evaluated the various important parameters for the production of these proteins. Finally, we analyzed and compared the functional properties of these proteins by tartrate resistant acid phosphatase (TRAP) assay and quantitative real-time polymerase chain reaction (qRT-PCR).

## Materials and Methods

### Reagents

Restriction enzymes were purchased from Takara (Shiga, Japan). All other chemicals used were purchased from Sigma- Aldrich (St. Louis, MO, USA) unless otherwise stated.

### Bacterial strains, vectors and media

*Escherichia coli* (*E. coli*) DH5α (Invitrogen, USA) was used for DNA manipulation. For preliminary screening experiments, *E. coli* BL21 (DE3) (Stratagene, USA), *E. coli* BL21 (Stratagene, USA), TOP10F'*E. coli* (Invitrogen, USA) and SHuffle Express *E.coli* (New England Biolabs, UK) cells were tested for expression of proteins. To optimize the production of both forms of RANKL, SHuffle Express *E. coli* was used as expression host. All *E. coli* strains were grown at 37°C in Luria-Bertani (LB) Broth (Becton, Dickinson and Company, USA) or LB agar plate supplemented with ampicillin antibiotic (100 µg/ml) when required. While pGEM-T Easy vector (Promega, USA) was used for the cloning of PCR products, pMAL-c5X vector (New England Biolabs, UK), pET32a (+) vector (Novagen, USA), and pGEX-5X-1 (GE Healthcare, UK) were used as expression vectors.

### Cell line and culture condition

The mouse macrophage cell line RAW 264.7, a well-established osteoclastogenic cell system that differentiate into TRAP-positive functional osteoclasts when co-cultured with RANKL [35], was purchased from American Type Culture Collection (ATCC, USA). RAW 264.7 cells were maintained in Dulbecco's modified Eagle's medium (Thermo Scientific HyClone, USA) supplemented with 10% fetal bovine serum (Thermo Scientific HyClone, USA) and antibiotics (100 U/ml penicillin G and 100 µg/ml streptomycin) at 37°C with 5% CO_2_.

### Computational codon optimization, synthetic gene construction and amplification

The full-length mRNA sequence (951 bp) of transmembrane RANKL of *Mus musculus* (GenBank accession no. AF013170.1) was taken for codon optimization by DNAWorks (v3.2.2) software [36] (Table S1). Expected codon adaptation index (CAI) values of wild type and codon optimized mRANKL were calculated from E-CAI server (http://genomes.urv.es/CAIcal), using a predefined reference set of highly expressed *E. coli* genes. The codon optimized transmembrane RANKL gene (*mRANKL*), flanked by *Nde*I and *Sal*I restriction sites, was synthesized and provided as an *Nde*I/*Sal*I insert in pUCIDT vector by Mbiotech (Gyeonggi-Do, Korea). The synthetic *mRANKL* was excised by digesting with *Nde*I/*Sal*I, purified with gel extraction kit (NucleoGen, Korea) and then ligated downstream of *malE* gene encoding maltose binding protein (MBP) in pMAL-c5X expression vector using T4 DNA ligase (Takara, Japan) to obtain recombinant plasmid pOmR-c5X. The ligation products were transformed into *E. coli* DH5α competent cells (Invitrogen, USA) for plasmid amplification and selected on LB agar plates with 100 µg/ml ampicillin. The plasmid was extracted using DNA purification kit (NucleoGen, Korea) and the ligation was confirmed by restriction enzyme digestion and DNA sequencing.

### Cloning and construction of expression vectors

Polymerase chain reaction (PCR) was used to amplify *mRANKL* or optimized extracellular RANKL gene (*RANKL-Ex*) encoding for the extracellular domain (137–316 region) of full length mRANKL. PCR was performed in a Takara PCR thermal cycler (Takara, Japan) with a set of primers (Table 1) using AccuPower PCR PreMix containing ***Top*** DNA Polymerase (Bioneer, Korea) according to the manufacturer's instructions. PCR reactions were carried out in a total volume of 20 µl with 50 ng plasmid DNA and 10 pmole of each primer under the following conditions: 3 min denaturation at 94°C followed by 30 cycles of extension (30 s at 94°C, 30 s at 52°C, then 1 min at 72°C) and final extension of 7 min at 72°C. PCR products were purified with gel extraction kit and ligated into pGEM-T Easy vector using T4 DNA Ligase (Promega, USA). The ligation products were transformed into ***E. coli*** DH5α and selected on LB agar plates with 100 µg/ml ampicillin. The ligation was confirmed by plasmid DNA isolation, restriction enzyme digestion, and DNA sequencing.

**Table 1:** Primers used in this study.

Gene	GenBank ID	Sequences
***RANKL-Ex***	-	Foreward: 5′GTGCATATGCAACGTTTCTCTGGTGCT 3′ Reverse:5′CCGGTCGACGTCGATGTCCTGAACTTT 3′
***mRANKL***	-	Foreward:5′ TATCCCGGGATGCGTCGTGCGAGCCGC 3′ Reverse:5′CCGCTCGAGGTCGATGTCCTGAACTTT 3′
***Trap***	NM_007388	Foreward: 5′ GCGACCATTGTTAGCCACATACGG 3 Reverse: 5′ CGCCCAGGGAGTCCTCAGATCCAT 3′
***CalcR***	NM_001042725	Foreward: 5′ TGGTGCGGCGGGATCCTATAAGTT 3′ Reverse: 5′ GTGGATAATGGTTGGCACTATCGG 3′
***Cfms***	NM_001037859	Foreward: 5′ TGGATGCCTGTGAATGGCTCTGAT 3′ Reverse: 5′ ATGGGCAGCTGGCTCTGAATGATC 3′
***Ctsk***	NM_007802	Foreward:5′ GAAATCTCTCGGCGTTTAATTTGGGAG 3′ Reverse: 5′ GGGGTATAGAGAGTGTCATTACTGTAG 3′
***Nfatc1***	NM_001164109.1	Foreward: 5′ TCCTGCTCCTCCTCCTGCTGCTCG 3′ Reverse: 5′ GCTGCTGGCAAGGCAGAGTGTGCT 3′
***Gapdh***	XM_132897.1	Foreward: 5′ AACTTTGGCATTGTGGAAGGGCTC 3′ Reverse: 5′ AAGGCCATGCCAGTGAGCTTC 3′

Further, *mRANKL* ligated into pGEM-T Easy vector was excised with *Xma*I-*Xho*I and inserted into pGEX-5X-1 to obtain pOmR-G5X. The plasmid pOmR-c5X harboring *mRANKL* was amplified, cut and ligated into *Nde*I- *Sal*I sites of pET32a (+) to obtain pOmR-32. Likewise, *RANKL-Ex* was excised from its pGEM-T vector with *Nde*I/*Sal*I, and then ligated into pMAL-c5X to generate pOsREx-c5X. The ligation products were transformed into *E. coli* DH5α for plasmid amplification.

### Protein expression, isolation and analysis

To optimize the expression of protein in *E. coli*, different expression hosts were transformed with the recombinant expression plasmids (Table 2). The transformed single colony was inoculated in 4 ml of LB medium supplemented with 100 µg/ml of ampicillin and incubated overnight at 37°C. 500 µl of overnight culture was used to inoculate 100 ml of the same medium and incubated at 37°C. When the culture reached an OD_600_ of 0.5–0.7, expression of protein was induced by either 0.4 mM IPTG or 20 mM lactose and then incubated at 30 °C for 6 h. The cells were harvested by centrifugation at 3,000 rpm for 10 min, washed twice with ice-cold PBS buffer and resuspended in 2 ml of following buffers related to protein-tagged system such as column buffer (20 mM Tris-HCl, 200 mM NaCl, 1 mM EDTA, pH 7.4) for MBP-tagged system, His_6_-binding buffer (20 mM Tris-HCl, 5 mM imidazole, 0.5 mM NaCl, pH 7.9) for His_6_-tagged system and GST-binding buffer (10 mM NaH2PO4, 1.8 mM KH2PO4, 2.7 mM KCl, 140 mM NaCl, pH 7.3) for GST-tagged system, followed by 10 min incubation on ice. The cells were then disrupted by sonication (Vibra Cell; Sonics & Materials, Newtown, USA) in a cycle of 9 s pulses and 4 s standbys for a total of 8 min in an ice bath. The lysate was cleared by centrifugation (12,000×**g**) for 30 min at 4°C. Expression of protein was monitored in 4–20% SDS gel (Komabiotech, Korea) using sodium dodecyl sulfate-polyacrylamide gel electrophoresis (SDS-PAGE).

**Table 2:** Bacterial strains and plasmids used in this study.

E. coli host	Vector system	Recombinant plasmid	Expression system	Induction	Expression of mRANKL
E. coli BL21 (DE3)	pMAL-c5X	pOmR-c5X	E. coli BL21 (DE3)- pOmR-c5X	IPTG/Lactose	Too low
E. coli BL21	pMAL-c5X	pOmR-c5X	E. coli BL21- pOmR-c5X	IPTG/Lactose	Too low
TOP10F'E. coli	pMAL-c5X	pOmR-c5X	TOP10F'E. coli-pOmR-c5X	IPTG/Lactose	Too low
Shuffle Express E. coli	pMAL-c5X	pOmR-c5X	Shuffle E. coli-pOmR-c5X	IPTG	Too low
Shuffle Express E. coli	pMAL-c5X	pOmR-c5X	Shuffle E. coli-pOmR-c5X	Lactose	Significant
E. coli BL21 (DE3)	pET32a(+)	pOmR-32	E. coli BL21 (DE3)- pOmR-32	IPTG/Lactose	ND
E. coli BL21	pET32a(+)	pOmR-32	E. coli BL21- pOmR-32	IPTG/Lactose	ND
TOP10F'E. coli	pET32a(+)	pOmR-32	TOP10F' E. coli-pOmR-32	IPTG/Lactose	ND
Shuffle Express E. coli	pET32a(+)	pOmR-32	Shuffle E. coli-pOmR-32	IPTG/Lactose	ND
E. coli BL21 (DE3)	pGEX-5X-1	pOmR-G5X	E. coli BL21 (DE3)- pOmR-G5X	IPTG/Lactose	ND
E. coli BL21	pGEX-5X-1	pOmR-G5X	E. coli BL21- pOmR-G5X	IPTG	Negligible
E. coli BL21	pGEX-5X-1	pOmR-G5X	E. coli BL21- pOmR-G5X	Lactose	ND
TOP10F'E. coli	pGEX-5X-1	pOmR-G5X	TOP10F' E. coli-pOmR-G5X	IPTG/Lactose	ND
Shuffle Express E. coli	pGEX-5X-1	pOmR-G5X	Shuffle E. coli-pOmR-G5X	IPTG/Lactose	ND
Shuffle Express E. coli	pMAL-c5X	pOsREx-c5X	Shuffle E. coli-pOsREx-c5X	Lactose	Significant

### Response surface methodology for optimization of protein expression

Central composite design (CCD) and response surface methodology (RSM) were employed to optimize the culture conditions for the production of maltose binding protein-tagged mRANKL in SHuffle Express *E.coli*. A 2^4^ full factorial central composite rotary design for four independent variables with replicates at the center point and star points were used in this study. The variables; optical density before induction (OD_600_) (0.5–0.7), lactose concentration (5–10 mM), post-induction temperature (22–30°C) and post-induction time (4–6 h) were tested using a statistical analysis and RSM each at five coded levels (−α, −1, 0, +1, +α) as shown in Table 3. The central composite design at the given range of the above mentioned parameters in terms of codes is shown in Table 4. A total of 30 experimental trials including 16 trials for factorial design, eight trials for axial points and six trials for replication of the central points were performed. The experimental data obtained were analyzed statistically by regression analyses using the following second-order polynomial equation: 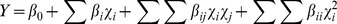
(1)where Y is the predicted response (mRANKL (mg/L)) used as a dependent variable; n is the number of independent variables (factors), *x_i_* (i = 1, 2) is the input independent variable (factors); *β_0_*, a constant coefficient, is the value of the fixed response at the center point of the design, and *β_i_*, *β_ij_* and *β_ii_* are the coefficients of linear, interaction and quadratic regression terms, respectively. The statistical software package, Design-Expert 8.0.7.1 (Stat-Ease, Inc., Minneapolis, USA) was used for regression analysis of experimental data and to plot the 3D surface response models. The statistical analysis of the model was represented in the form of analysis of variance (ANOVA) and the optimal points of the variables were obtained by “point optimization” tool of Design Expert.

**Table 3:** The independent variables and coded values of variables used in central composite rotary design.

Independent variables	Units	Levels				
		-α	-1	0	1	α
Cell density (OD_600_)	-	0.4	0.5	0.6	0.7	0.8
Lactose	mM	2.5	5	7.5	10	12.5
Temperature	°C	18	22	26	30	34
Induction time	h	3	4	5	6	7

**Table 4:** CCD with measured and predicted response of mRANKL production.

Run	A OD600	B Lactose (mM)	C Temperature (°C)	D Induction time (h)	Response mRANKL (mg/L)	
					Predicted value	Actual value
1	−1	−1	−1	−1	16.7	15.9
2	1	−1	−1	−1	17.3	16.3
3	−1	1	−1	−1	16.8	17.5
4	1	1	−1	−1	26.8	27.9
5	−1	−1	1	−1	26.1	26.8
6	1	−1	1	−1	16.1	19.2
7	−1	1	1	−1	24.7	23.1
8	1	1	1	−1	23.9	23.9
9	−1	−1	−1	1	39.7	40.5
10	1	−1	−1	1	23.7	27.4
11	−1	1	−1	1	37.8	36.8
12	1	1	−1	1	31.1	31.3
13	−1	−1	1	1	41.8	42.8
14	1	−1	1	1	15.1	15.3
15	−1	1	1	1	38.3	40.2
16	1	1	1	1	20.9	23.8
17	−2	0	0	0	33.5	34.3
18	2	0	0	0	16.8	13.3
19	0	−2	0	0	39.1	36.9
20	0	2	0	0	45.1	44.6
21	0	0	−2	0	11.5	11.3
22	0	0	2	0	10.8	8.3
23	0	0	0	−2	15.7	16.2
24	0	0	0	2	35.8	32.6
25	0	0	0	0	49.5	49.7
26	0	0	0	0	49.5	50.6
27	0	0	0	0	49.5	48.7
28	0	0	0	0	49.5	47.8
29	0	0	0	0	49.5	48.9
30	0	0	0	0	49.5	51.6

### Purification of soluble proteins and molecular weight determination

The soluble proteins were purified by amylose affinity chromatography according to the manufacturer's instructions. Briefly, crude protein extract in column buffer was loaded in 2 ml of amylose resin (New England Biolabs, UK) and washed with 12 column volumes of column buffer, and the proteins were eluted with 10 mM maltose in column buffer. Elution fractions were analyzed by SDS–PAGE followed by staining with Coomassie Brilliant Blue R-250. The purified MBP-tagged proteins were dialyzed against 20 mM Tris-HCl, 25 mM NaCl buffer (pH 7.4) at 4°C for 24 h with three buffer changes. Endotoxins were removed by Detoxi-gel endotoxin removing columns (Thermo Scientific Pierce, USA) according to the manufacturer's instructions. Protein concentrations were determined by measuring the absorbance at 280 nm using Nanophotometer (Implen GmbH, Germany).

Separation of RANKL from its MBP fusion partner was accomplished by proteolytic cleavage with Factor Xa (Amersham Biosciences, UK), and the reaction was performed overnight at 4°C in 50 mM Tris-HCl, 25 mM NaCl, 1 mM EDTA (pH 7.4). Uncleaved fusion protein and MBP were removed by second passage through amylose resin as described before. The protein was further purified by gel filtration chromatography using Superdex 20010/300 GL column (GE Healthcare, UK) equilibrated with buffer containing 20 mM Tris-HCl (pH 7.4). The column was connected to an AKTA explorer 100 fast protein liquid chromatography apparatus (Amersham Biosciences, UK) and eluted with the same buffer. Purified protein was run in gel electrophoresis under native condition using Tris-Glycine-PAG Pre-Cast Gel, non-SDS, 4–20% (Komabiotech, Korea) for mass estimation of native membrane protein. For molecular mass determination, 5 mg/ml of purified protein was applied to a Superdex 200 10/300 GL column. The column was calibrated with gel filtration molecular weight markers kit (MWGF200, Sigma). The gel-phase distribution coefficient (*K_av_*) was calculated as 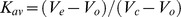
where *V_e_* is elution volume, *V_o_* is void volume and *V_c_* is column volume.

### Western blot analysis for confirmation of protein expression

Heterologous expression of the proteins was confirmed by western blot analysis. Briefly, the proteins were separated under reducing conditions in a 4–20% SDS gel using XCell ***SureLock*** Mini-Cell **(Life Technologies, USA)** at 130 V for 2 h. Precision plus protein dual-color standards (BioRad, USA) were used as molecular weight marker. After electrophoresis, the proteins were electro-transferred to nitrocellulose membranes (Protran nitrocellulose membrane, Whatman, UK) at 10 V for 60 min, using Trans-Blot SD Semi-Dry Electrophoretic Transfer Cell (Bio-Rad, USA). The membrane was blocked with 5% skim milk in Tris-buffered saline-Tween (TBST) buffer for 60 min at room temperature and then washed three times with TBS-Tween. The membrane was then incubated with an antibody against RANKL (R&D Systems, USA) on a shaker for overnight at 4 °C, followed by washing with TBST buffer three times for 15 minutes each. The membrane was then incubated with goat IgG horseradish peroxidase (HRP)-conjugated antibody (R&D Systems, USA) in TBST buffer for 1 hour at room temperature. After three successive washing with TBST buffer for 15 minutes each, the proteins were detected by enhanced chemiluminescence (ECL) detection system (GE Healthcare, UK) and exposed to **Gel Doc XR system** (BioRad, USA) to capture chemiluminescent signal on the western blot.

### Osteoclast differentiation assay

RAW 264.7 cells were seeded on 6-well plates at a density of 2×10^4^ cells per well in the presence of different concentration of mRANKL (30–100 ng/ml) or RANKL-Ex (100 ng/ml) for 6 days. The culture medium was replaced with fresh medium containing above mentioned samples every 48 h over the course of 6 days. The cultured cells were then subjected to TRAP-staining (B-Bridge, USA) according to the manufacturer's instructions to confirm the generation of TRAP-positive osteoclast-like cells. Briefly, the cells were washed with PBS and fixed with the fixative reagent for 5 min at room temperature. The cells were washed three times with distilled water and then stained with the chromogenic substrate for 60 min at 37°C and finally washed with distilled water to stop the reaction when optimum color was achieved. TRAP-positive osteoclasts were visualized by light microscopy and photographed. Untreated cells were used as controls. Each osteoclast differentiation assay was performed at least 3 times. In order to quantify the TRAP activity, 30 µL of culture supernatant was incubated at 37°C for 3 h in the presence of 170 µL of the chromogenic substrate diluted in tartrate-containing buffer (3 mg/5 mL). The absorbance was measured at 540 nm using Infinite 200 PRO multimode reader (Tecan, Switzerland).

### RNA extraction, reverse transcription and real-time PCR analyses

To determine the effect of RANKL in osteoclast differentiation, RAW 264.7 cells were treated with 100 ng/ml of mRANKL or RANKL-Ex. The level of transcription of the associated genes during osteoclast differentiation was then analyzed by isolating total RNAs from the RANKL-treated cells using Trizol reagent **(Life Technologies, USA)** following the manufacturer's instructions. Briefly, the cells were homogenized in 1 ml Trizol. Samples were mixed with 200 ml chloroform and then centrifuged 12,000×g for 15 minutes at 4°C. The upper aqueous phase was transferred carefully into fresh tube without disturbing the interphase and equal volume of isopropanol was added in the tube. Mixtures were thoroughly resuspended and centrifuged at 12,000×g for 10 minutes at 4°C. The precipitated RNA pellets were washed with 1 ml ethanol (75%, v/v). RNA pellets were recovered after centrifugation at 12,000×g for 5 min at 4°C. RNA samples were allowed to air-dry for 2–3 min and then resuspended in 50 µl diethyl pyrocarbonate-treated water **(Life Technologies, USA)**. RNA was further purified using a Qiagen RNeasy mini kit and Rnase-free DNAse set (Qiagen, Germany) according to the manufacturer's specifications. RNA was quantified using Nanophotometer. The equal amount of total RNA (1 µg) from each sample was reverse-transcribed to cDNA at 42°C for 30 minutes in a final volume of 20 µl, using Quantitect reverse transcription kit (Qiagen, Germany) according to the manufacturer's protocol. Each cDNA was stored at −20°C until use.

qRT-PCR was conducted using TOPreal qPCR 2X PreMIX (SYBR Green) (Enzynomics, Korea) in a total reaction volume of 20 µl. cDNA templates and primers were added to SYBR Green containing nTaq-HOT DNA polymerase, dNTP mixture and SYBR-Green I. Real-time PCR was then performed using MyiQTM single color real-time PCR detection system (Bio-Rad) under following conditions: 30 s at 95°C, followed by 40 cycles at 95°C for 10 s, 60°C for 30 s; then 72°C for 1 min and followed by a dissociation stage (95°C for 15 s). A melting curve was plotted to ensure the specificity of amplification products. The 2^−ΔΔCt^ method [37] was used to analyze the relative changes in the level of target gene transcription. The primers used for qRT-PCR are shown in Table 1.

## Results

### Codon-optimization and cloning of *mRANKL*

Different organisms use synonymous codons with different preferences. As a consequence, heterologous proteins, especially human proteins, fail to express in *E. coli* due to the presence of “rare” codons in the target mRNA that are infrequently used by *E. coli*. This includes the codons for arginine (AGA, AGG, CGA), isoleucine (AUA), leucine (CUA), and proline (CCC). One can improve the expression of heterologous proteins significantly in *E. coli* by selecting the synonymous codons that are favored by the *E. coli* host. The full-length sequence of *mRANKL* of *Mus musculus* possesses 19 rare codons including 11 codons for arginine (AGA, AGG, CGA), 3 codons for isoleucine (AUA), 2 codons for leucine (CUA), and 3 codons for proline (CCC) as predicted by the rare codon calculator, RaCC (http://nihserver.mbi.ucla.edu/RACC/). DNAWorks (v3.2.2) was used to modify 198 of the 316 codons with synonymous codons predicted to occur frequently in highly expressed genes in *E. coli*. Thus, *mRANKL* was codon optimized while maintaining the integrity of the native amino acid structure (Table S1). CAI was used to estimate the adaptation of codon optimized mRANKL to host codons. CAI is a measurement of the relative adaptiveness of the codon usage of a gene towards the codon usage of highly expressed genes in the host. The codon optimization of mRANKL increased the CAI value [38] from 0.64 (wild type) to 0.76 (codon optimized) indicating that the optimized gene was closer to the highly expressed genes in *E. coli*. Finally, *mRANKL* gene was synthesized and cloned into different expression vectors as described in the materials and method section.

### Expression of mRANKL in *E. coli*

On the basis of ‘trial and error’ approach, three vector systems, pET32a(+), pGex-5X-1 and pMAL-c5X with three different solubility tags, i.e., hexahistidine (His_6_), glutathione S-transferase (GST) and maltose-binding protein (MBP), respectively, and four *E. coli* hosts including *E. coli* BL21 (DE3), *E. coli* BL21, TOP10F' *E. coli* and SHuffle Express *E. coli* were selected to test the heterologous expression of mRANKL. Accordingly, *E. coli* MBP-mRANKL, *E. coli* His_6_-mRANKL and *E. coli* GST-mRANKL expression systems were generated and recombinant RANKL was extracted as MBP-mRANKL, His_6_-mRANKL and GST-mRANKL from the respective system with IPTG or lactose as inducer. The expression of mRANKL in these systems was monitored by SDS-PAGE. There was no visible expression of mRANKL in SDS gel from all *E. coli* hosts with His_6_-tagged expression system and three *E. coli* hosts with GST-tagged system in all conditions tested. A negligible amount of soluble GST-mRANKL was obtained in *E. coli* BL21 when IPTG was used as inducer (data not shown). While three *E. coli* hosts with MBP-tagged system gave too low yield of mRANKL in all conditions tested, the significant amount of mRANKL was produced in SHuffle *E.coli*-pOmR-c5X where the high level expression of mRANKL was obtained in inclusion bodies when lactose was used as inducer (data not shown). These preliminary experiments showed that the highest protein yield was obtained from SHuffle *E.coli*-pOmR-c5X among the systems tested and thus, this system was further employed to optimize the production of soluble mRANKL in *E. coli*.

### Optimization of mRANKL expression using response surface methodology

Besides appropriate expression vector, fusion tag and expression host, there are multiple parameters that can be varied when optimizing an expression of target protein in *E. coli*, with each parameter affecting the solubility and activity of the protein. Due to several parameters, RSM was applied to determine optimal conditions for the production of mRANKL from *E. coli*. RSM is a statistical method useful for analyzing the effect of several independent variables influencing the responses by varying them simultaneously with limited number of experiments. A central composite design (CCD), an efficient design tool for fitting second-order models under RSM, was used to investigate the effect of four most important variables such as cell density before induction, lactose concentration, post-induction temperature and post-induction time that influence the heterologous production of most recombinant proteins. In the optimization procedure, the response of the statistically designed combinations was determined, the coefficients by fitting the experimental data to the response functions were estimated, the response of the fitted model was predicted and the adequacy of the model was verified using software Design-Expert (version 8.0.1). A list of independent variables and coded factor levels are given in Table 3. A 2^4^ full factorial CDD design matrix and the predicted and experimental results of mRANKL production are given in Table 4. A total of 30 experiments were conducted for four factors at five levels to optimize the production of mRANKL. The number of experiments required (N) is predicted by the expression: 2^k^ (2^4^ = 16; star points) +2 k (2×4 = 8; axial points) +6 (center points; 6 replications). Thirty observed responses were used to compute the model using the least square method. The response (mRANKL (mg/L) was correlated with the four different factors, cell density, lactose concentration, post-induction temperature and post-induction time, as a function of the second-order polynomial equation, as represented by Eq. (1). From the experimental data, following quadratic regression model was obtained in coded form: 
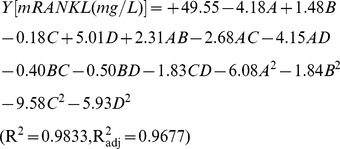
(2)where Y is the response (mRANKL production), and A, B, C and D are the coded terms for the four variables i.e. cell density before induction, lactose concentration, post-induction temperature and post-induction time respectively. The coefficients in front of A, B, C or D represent the effects of that particular factor, while the coefficients in front of AB, AC, AD, BC, BD or CD and those in front of A^2^, B^2^, C^2^ or D^2^ represent the interaction between the two factors and the quadratic effects, respectively. The positive sign indicates a synergistic effect, while the negative sign indicates an antagonistic effect.

Different combinations of four variables yielded MBP-tagged mRANKL production ranging from 8.3 to 51.6 mg/L as illustrated in Table 4. The maximum production of fusion mRANKL (51.6 mg/L) was obtained at central point values with OD_600_: 0.6, lactose concentration: 7.5 mM, post-induction temperature: 26°C and post-induction time: 5 h, respectively and the lowest production was found at OD_600_: 0.6, lactose concentration: 7.5 mM, post-induction temperature: 34°C and post-induction time: 5 h, respectively.

### Validation of the model

**Figure 1:**
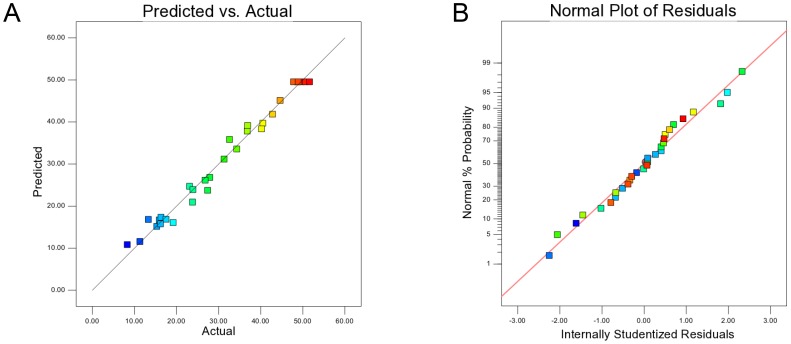
Diagnostic plots for estimating the adequacy of the regression model. Correlation between predicted and actual value for mRANKL production (A). The studentized and normal percentage probability plot of mRANKL production (B).

The results of analysis of variance (ANOVA) values for the quadratic regression model obtained from CCD employed in the optimization of mRANKL production are given in Table 5. The statistical significance of a quadratic model was tested through F- and p-values for analysis of variance. The large F-value indicates that most of the variation can be explained by a regression equation whereas a low p-value (<0.05) indicates that the model is considered to be statistically significant [34]. Thus, the high F-value (63.23) and very low probability of p > F value (0.0001) obtained revealed that the regression is statistically significant. Moreover, the results of the lack of fit test for the models showed that the lack of fit is not statistically significant at 95% confidence level. The adequacy of quadratic model can be confirmed by the coefficient of determination R^2^ along with an acceptable agreement with the adjusted determination coefficient R^2^_adj_. The ANOVA analysis reported the high R^2^ value of 0.9833 and R^2^_adj_ value of 0.9677 for mRANKL production, both of which are close to 1, ensuring a high correlation between the experimental values and the predicted values. The diagnostic plots used for estimating the adequacy of the regression model are shown in Figure 1. From the correlation between the actual and the predicted values of mRANKL production, it is evident that there are tendencies in the linear regression fit, and the model adequately explains the experimental range studied (Figure 1A). The actual value is the result obtained for a specific run and the predicted value is obtained from the independent variables in the CCD model. The data points in the normal percentage probability and studentized residual plot indicated that neither response transformation was required nor there was any apparent problem with normality (Figure 1B). Thus, ANOVA results indicated the satisfactory adjustment of the quadratic models to the experimental data.

**Table 5:** ANOVA for response surface quadratic model for mRANKL production.

Source	df	Sum of Squares	Mean Square	F- Value	p-value Prob > F
Model	14	5218.066	372.719	63.23285	<0.0001
A-OD_600_	1	420.8438	420.8438	71.39735	<0.0001
B-Lactose	1	53.10375	53.10375	9.009204	0.0089
C-Temperature	1	0.84375	0.84375	0.143145	0.7105
D-Induction time	1	603.0038	603.0038	102.3013	<0.0001
AB	1	86.02563	86.02563	14.59449	0.0017
AC	1	115.0256	115.0256	19.51443	0.0005
AD	1	276.3906	276.3906	46.89046	<0.0001
BC	1	2.640625	2.640625	0.44799	0.5135
BD	1	4.100625	4.100625	0.695683	0.4173
CD	1	53.65562	53.65562	9.102831	0.0087
A∧2	1	1016.091	1016.091	172.3827	<0.0001
B∧2	1	93.7686	93.7686	15.90811	0.0012
C∧2	1	2520.691	2520.691	427.6424	<0.0001
D∧2	1	966.625	966.625	163.9907	<0.0001
Residual	15	88.41583	5.894389		
Lack of Fit	10	78.88083	7.888083	4.136383	0.0653
Pure Error	5	9.535	1.907		

A  =  OD_600_; B  =  Lactose concentration; C  =  Temperature; D  =  Induction time; df  =  Degrees of freedom.

### Interactive effect of process independent variables

**Figure 2:**
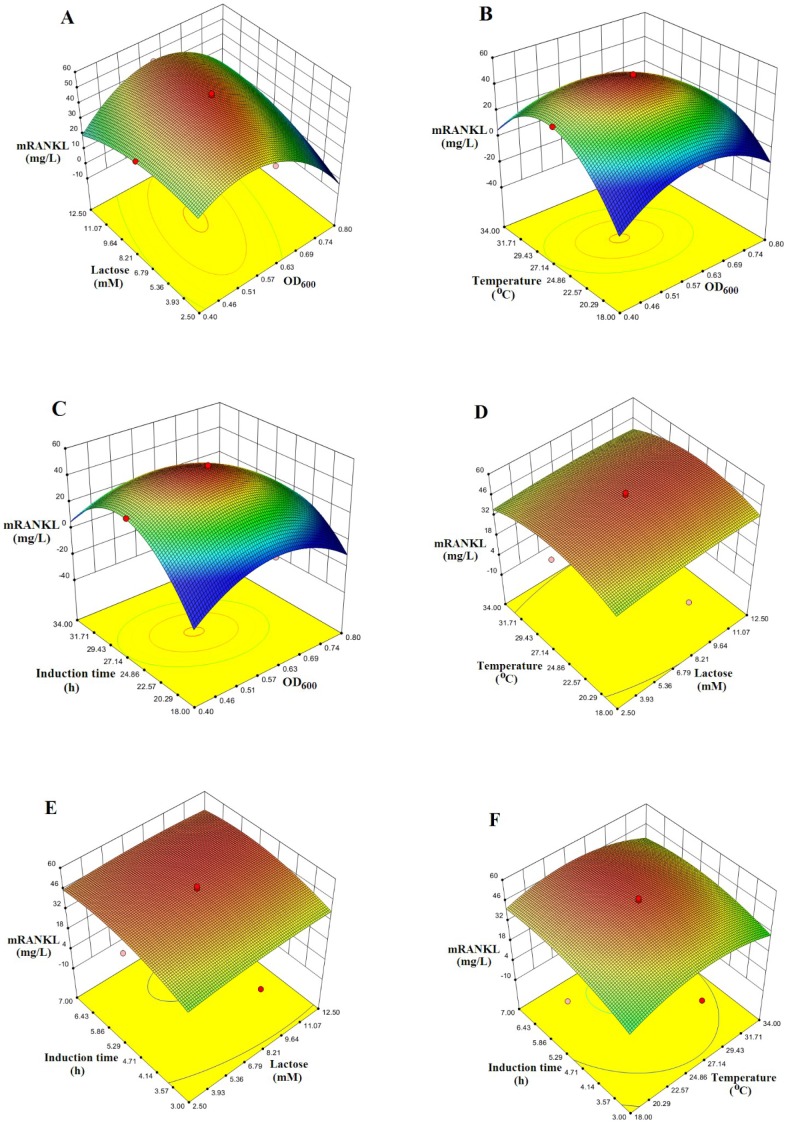
Response surface plots to visualize the relationship between the response and experimental levels of each factor. Three dimensional surface plots of combined effect of cell density (OD_600_) and lactose concentration (A), OD_600_ and post-induction temperature (B), OD_600_ and post-induction time (C), post-induction temperature and lactose concentration (D), post-induction time and lactose concentration (E) and post- induction temperature and post-induction time (F) on mRANKL production.

The interactive effects of variables on production of mRANKL were obtained in the form of three dimensional (3D) response surface plots as shown in Figure 2 (A-F). The response surface plots are the graphical representation of the regression equation used to visualize the relationship between the response and experimental levels of each factor. The integrated effect of lactose and cell density before induction on mRANKL production is shown in Figure 2A. The optimum conditions for mRANKL production was found to be at OD_600_ of 0.6 and lactose concentration of 7.5 mM. While, increased production of mRANKL was observed with increasing OD_600_, the lactose concentration showed the less effect on mRANKL production. However, an increase in OD_600_ beyond the optimum region resulted in a decreased production of mRANKL. Figure 2B indicates the production of mRANKL as a function of post-induction temperature and OD_600_. The production of mRANKL was increased with increasing temperature and OD_600_. At the temperatures higher than 30°C, the production of mRANKL began to decrease at all OD_600_. Figure 2C shows correlation of post-induction time and OD_600_ with mRANKL production. It can be observed that increase in mRANKL production occurred with both OD_600_ and post-induction time. An increase in both factors beyond the optimum region (OD_600_: 0.6 and induction time: 5 h) resulted in a decrease in mRANKL production. Figure 2D indicates the interactive influence of post-induction temperature and lactose concentration on the extent of mRANKL production. While the integrated impact of post-induction time and lactose concentration on mRANKL production is depicted in Figure 2E, the combined impact of induction time and post-induction temperature is shown in Figure 2F. Thus, these plots showed that OD_600_ lesser than 0.7, post-induction time of 5 h and lower post-induction temperature values were favorable for RANKL production, while concentration of lactose showed lower impact on mRANKL production.

### Confirmation experiments at optimum conditions

**Figure 3:**
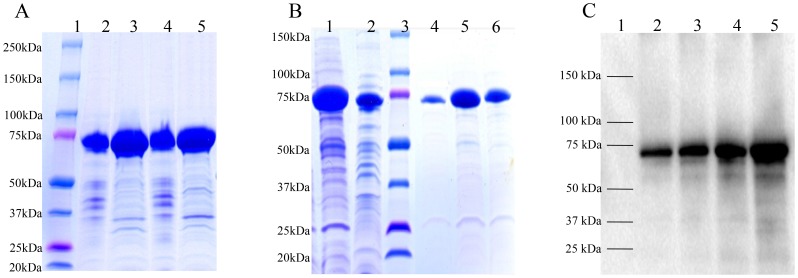
SDS-PAGE and Western blot analyses of mRANKL fusion proteins. A: Comassie blue stained SDS gel showing lysates from two SHuffle *E. coli*-pOmR-c5X colonies expressing the mRANKL fusion proteins (∼75 kDa). Lane 1: Protein standards, molecular masses are indicated in kilodaltons (kDa); Lanes 2 and 3: Insoluble and soluble fractions from clone 1, respectively; Lanes 4 and 5: Insoluble and soluble fractions from clone 2, respectively. B: Comassie blue stained SDS gel showing purification of mRANKL using amylose resin. Lane 1: crude fusion mRANKL; Lane 2: Flow through; Lane 3: Protein standards, molecular masses are indicated in kilodaltons (kDa); Lanes 4–6: Elution fractions. C: Western blot analysis of mRANKL. Different amounts of purified fusion mRANKL was run in 4–20% SDS gel and transferred onto a nitrocellulose membrane. Detection was performed with anti-RANKL primary antibody, goat IgG HRP-conjugated secondary antibody and chemiluminescent substrate. Lane 1: Protein marker; Lanes 2–5: 30, 50, 100, and 200 ng of purified fusion mRANKL protein respectively.

**Figure 4:**
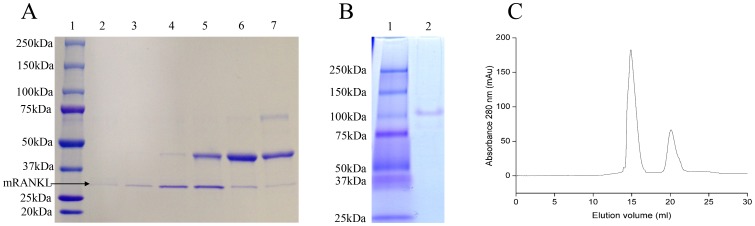
Purification of mRANKL by affinity chromatography and gel filtration chromatography. A.SDS-PAGE analysis of purified mRANKL. The fusion mRANKL was cleaved by Factor Xa, purified by affinity chromatography followed by gel filtration chromatography. Eluted proteins fractions (Lane 2–7) were checked by SDS-PAGE gel. Lane 1: Protein standards, molecular masses are indicated in kilodaltons (kDa). B. Native PAGE analysis. PAGE analysis demonstrated the native state of mRANKL (molecular weight of approximately 100 kDa), consistent with the size of a homotrimer. Lane 1: Protein standard; Lane 2: mRANKL. C. Gel filtration chromatography profile. The elution peaks at 14.90 ml and 20.09 ml correspond to estimated molecular masses of ∼97 and ∼44 kDa, based on the elution volumes of known molecular standards. The estimated molecular masses are close to the predicted molecular weight of trimeric form (∼104 kDa) and monomeric form of the protein (∼35 kDa). The y axis indicates the absorption at 280 nm in milliabsorbance units (mAU) and the x axis indicates the volume in milliliters (ml) passed over the column.

The agreements of the results obtained from the model and experiments were further confirmed by additional experiments by applying optimum conditions. The model predicted a maximum of 49.5 mg/L mRANKL production with a cell density before induction (OD_600_) of 0.6, lactose concentration of 7.5 mM, post-induction temperature of 26°C and post-induction time of 5 h. As shown in Table 6, mRANKL obtained from the additional experiments was found to be close to that predicted by the model. Experiments were performed in triplicate. The production of mRANKL was successfully scaled up from 100 ml to 1 L culture volume using these optimum conditions. Expression of fusion mRANKL (∼75 kDa) was assessed by SDS-PAGE (Figure 3A). The fusion protein was purified by amylose affinity chromatography (Figure 3B), and then confirmed by western blot analysis (Figure 3C). Finally, mRANKL was separated from MBP fusion partner and purified by affinity chromatography and gel filtration chromatography (Figure 4A).

**Table 6:** Confirmation experiments for mRANKL production.

	Expression condition				mRANKL (mg/L)
	OD_600_	Lactose (mM)	Temperature (°C)	Induction time (h)	
Predicted value	0.6	7.5	26	5	49.5
Experimental value	0.6	7.5	26	5	52.4

### Optimization of RANKL-Ex expression using response surface methodology

The successful expression of mRANKL in soluble form in SHuffle Express *E. coli* prompted us to overexpress RANKL-Ex. For this, *RANKL-Ex* was amplified and cloned to construct recombinant expression vector pOsREx-c5X, and transformed into SHuffle Express *E.coli*. CCD and RSM were employed to determine the optimum conditions for overexpression of RANKL-Ex in *E. coli*. Cell density before induction, lactose concentration, post-induction temperature and post-induction time were chosen as four independent variables. The experimental results were analyzed using Design Expert 8.0.7.1 and the regression model was proposed.

A list of independent variables and coded factor levels are given in Table 3. Accordingly, a total of 30 experiments were conducted for four factors at five levels to optimize the production of RANKL-Ex as shown in Table S2. The second-order polynomial model that characterizes the relationship between RANKL-Ex production and variables, is represented by Eq. (3). 
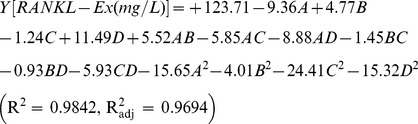
(3)where Y is the response (RANKL-Ex production), and A, B, C and D are the coded terms for the four variables, i.e., OD_600_ before induction, lactose concentration, post-induction temperature and post-induction time, respectively.

Different combinations of four variables yielded MBP-tagged RANKL-Ex production ranging from 19.9 to 128.7 mg/L as shown in Table S2. As in the case of fusion mRANKL, the maximum production of fusion RANKL-Ex (128.7 mg/L) was obtained at central point values with OD_600_: 0.6, lactose concentration: 7.5 mM, post-induction temperature: 26°C and post-induction time: 5 h, respectively, and the lowest production (19.9 mg/ml) was found at OD_600_: 0.6, lactose concentration: 7.5 mM, post-induction temperature: 34°C and post-induction time: 5 h, respectively, indicating that post-induction temperature above 30°C is not favorable for production of RANKL-Ex as well.

The results of analysis of variance (ANOVA) values for the quadratic regression model obtained from CCD employed in the optimization of RANKL-Ex production are given in Table S3. Thus, the high F-value (66.84), very low probability of p > F value (0.0001), R^2^ value of 0.9842 and R^2^_adj_ value of 0.9694 revealed the adequacy of quadratic model. The diagnostic plots used for estimating the adequacy of the regression model are shown in Figure S1 (A–B). The 3D response surface plots depicted in Figure S2 (A–F) represent the interactive effects of variables on production of RANKL-Ex within the experimental ranges. Additional confirmation experiments showed that the production of RANKL-Ex was found to be close to that predicted by the model under same conditions (Table S4). The production of RANKL-Ex was scaled up to 1 L culture media. Purified fusion RANKL-Ex (∼62 kDa) was assessed by SDS-PAGE and confirmed by western blot analysis as depicted in Figure S3 (A, B and C). Finally, RANKL-Ex was cleaved to remove its fusion partner and RANKL-Ex was further purified by affinity chromatography and gel filtration chromatography (data not shown).

### mRANKL self-assembles into homotrimer

Based on the crystallographic studies that extracellular RANKL self-associates as a homotrimer (9), self-assembly of mRANKL was examined by native polyacrylamide gel electrophoresis (PAGE) and gel filtration chromatography. Proteins run on PAGE in the absence of SDS will separate on the basis of their native forms enabling for mass estimation of native membrane proteins. PAGE analysis of purified mRANKL under native conditions demonstrated that it migrated with a molecular weight of approximately 100 kDa, consistent with the size of a homotrimer (Figure 4B). Similarly, purified mRANKL was applied on to Superdex 200 10/300 GL column as described in materials and methods. The resulting chromatogram exhibited two major peaks at 14.9 ml and 20.0 ml of elution volume corresponding to calculated molecular weight of approximately 97 kDa and 44 kDa (Figure 4C), based on the elution volumes of known molecular standards. The calculated molecular weights are close to the predicted molecular weight of trimeric form (∼104 kDa) and monomeric form of mRANKL (∼35 kDa).

### mRANKL induces osteoclast differentiation

**Figure 5:**
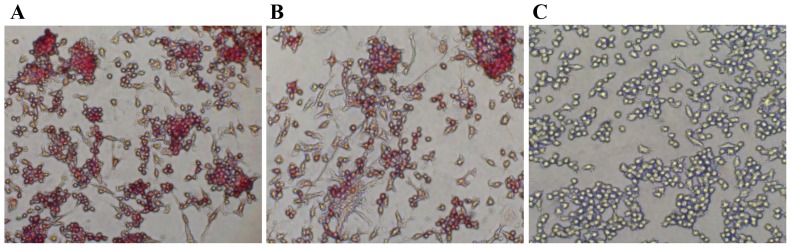
TRAP staining assay. RAW 246.7 cells were treated with each protein at the concentration of 100/ml for 6 days and TRAP-positive osteoclasts were visualized under microscope. mRANKL (A); RANKL-Ex (B); Untreated RAW 246.7 cells (C).

**Figure 6:**
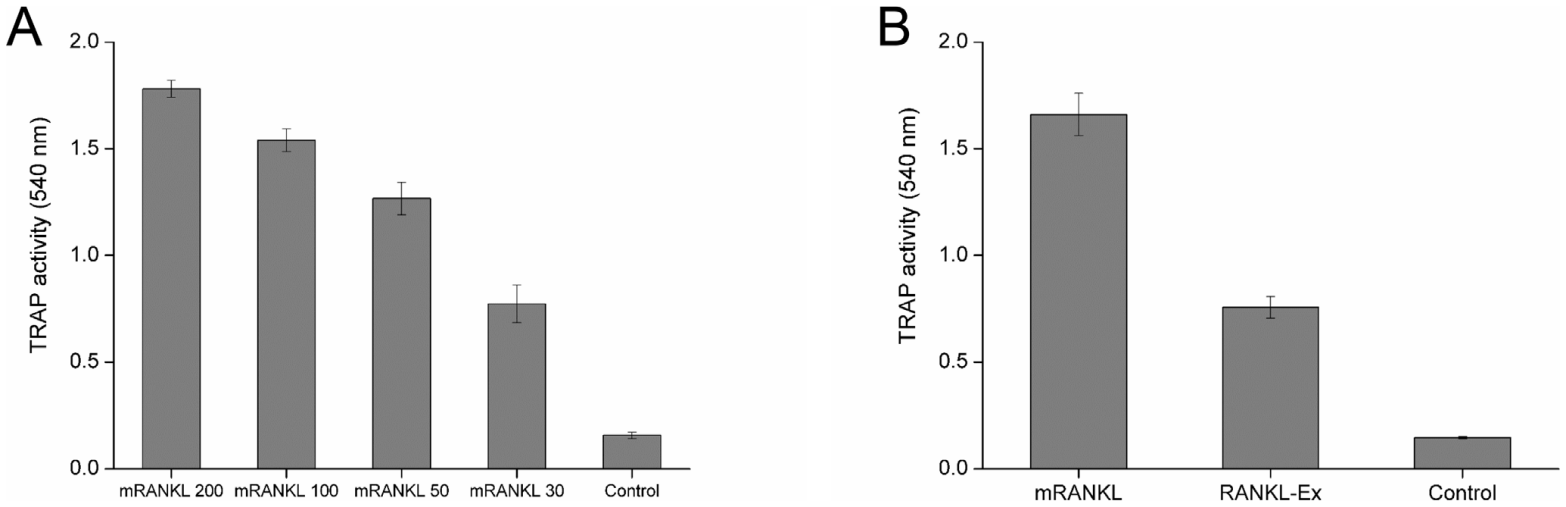
Induction of osteoclastogenesis by RANKL in RAW264.7 cells. TRAP activity was determined in the medium of RAW264.7 cells treated with mRANKL (30–200 ng/ml) for 6 days, numerical value with mRANKL denotes the concentration of mRANKL used (A); RAW264.7 cells were treated with 100 ng/ml of each mRANKL and RANKL-Ex for 6 days (B).

It has been well established that RANKL is the key cytokine that is essential for osteoclast differentiation/activation. RAW264.7 cell line is a model system that is being used extensively in *in vitro* osteoclast differentiation studies where osteoclasts are identified by TRAP assay. Since TRAP is expressed in high levels in the osteoclasts, it is used as a marker of osteoclast function [39]. Thus, to examine the potency of mRANKL to induce osteoclastogenesis *in vitro*, RAW264.7 cells were cultured at low cell density and treated with various concentrations of mRANKL for 6 days in order to promote cell differentiation. Cultures were then fixed and stained for TRAP activity as described in materials and method section. Untreated cultures are used as controls. After 6 days of culture, TRAP-positive osteoclasts were observed in the cultures treated with mRANKL when visualized under microscope (Figure 5). mRANKL at a concentration as low as 30 ng/ml was sufficient to induce TRAP release in RAW 264.7 cells. The TRAP activity increased significantly in the medium of RAW264.7 cells treated with mRANKL as compared to untreated cells. Moreover, mRANKL induced osteoclast differentiation in a concentration-dependent manner as evidenced by increasing TRAP formation in RAW264.7 cells (Figure 6A).

### mRANKL upregulates osteoclast-associated genes

**Figure 7:**
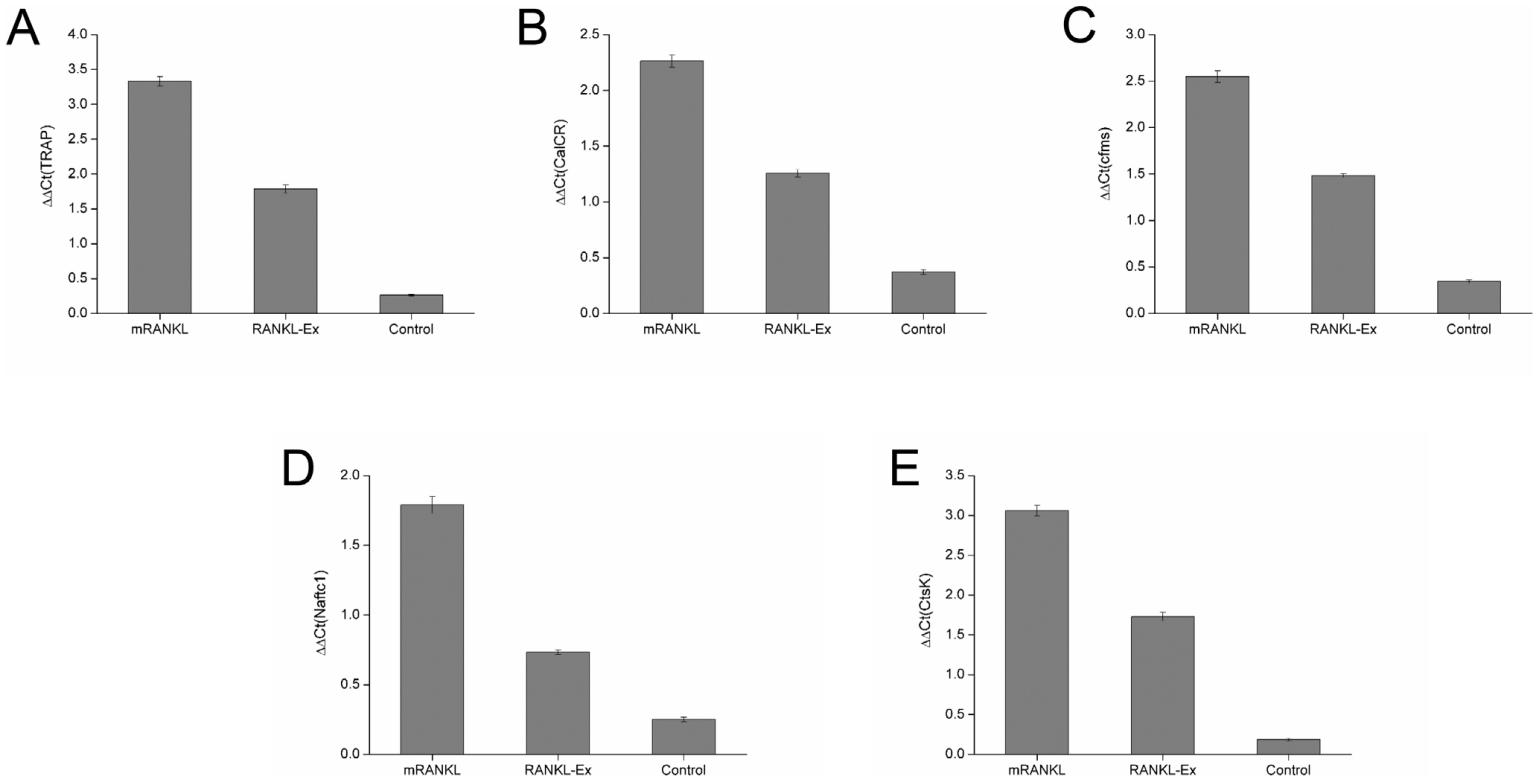
Quantitative RT-PCR analyses. The expression of osteoclast differentiation (A–C) and functional markers (D, E) were studied at day 6 after mRANKL (100 ng/ml) and RANKL-Ex (100 ng/ml) exposure to RAW 246.7 cells. The results of the mRNA levels of genes of interest were normalized to GAPDH (control gene) and plotted. The data shown are representatives of three different experiments.

During osteoclastogenesis, osteoclasts express several marker genes, such as *Trap*, *CalcR*, *Cfms*, *Nfatc1* and *CtsK*. These specific osteoclast genes **are the key indicators of** the differentiation of osteoclast precursor into osteoclasts. Therefore, the regulation of transcription of these genes, by the treatment of mRANKL in RAW 264.7 cells, were examined by qRT-PCR. When the transcription levels were quantitatively analyzed, ***Trap*** mRNA levels were approximately 15-fold higher in cells treated with mRANKL when compared to that of untreated cells (Figure 7A). Similarly, ***CalcR*** (Figure 7B) and *Cfms* (Figure 7C) mRNA expression levels were about 6-fold and 7.5-fold higher, respectively, in mRANKL treated cells. Likewise, *Nfatc1* (Figure 7D) ***CtsK*** (Figure 7E) mRNA levels were approximately 6-fold and 14-fold higher, respectively, by the treatment of mRANKL as compared to that of untreated cells.

### mRANKL is functionally superior to RANKL-Ex

To compare the functional difference between mRANKL and RANKL-Ex to induce osteoclastogenesis, RAW264.7 cells were treated with 100 ng/ml of mRANKL or RANKL-Ex for 6 days, and TRAP assay was performed as described in materials and method. It was found that secreted TRAP activity was approximately double in the medium of RAW264.7 cells treated with mRANKL as compared to that treated with RANKL-Ex (Figure 6B). Thus, the TRAP assay results showed that mRANKL is more efficient to induce osteoclast formation as compared to RANKL-Ex. Further, the effects of mRANKL and RANKL-Ex on mRNA levels of osteoclast phenotypic and functional markers were compared by qRT-PCR. ***Trap*** mRNA levels were approximately 2-fold higher in cells treated with mRANKL as compared to that of RANKL-Ex (Figure 7A). The mRNA levels of ***CalcR*** (Figure 7B) and *Cfms* (Figure 7C) were about 1.7-fold and 1.6-fold higher, respectively, in mRANKL treated cells. Similarly, the mRNA levels of *Nfatc1* (Figure 7D) and ***CtsK*** (Figure 7E) were induced approximately 2.5-fold and 1.6-fold higher, respectively, by the treatment of mRANKL as compared to that of RANKL-Ex.

## Discussion

In recent years, numerous studies have revealed the important roles of RANKL. But, most of the studies on physiological role of RANKL have been carried out with extracellular domain of RANKL. The extracellular RANKL is derived by proteolytic cleavage of mRANKL, a similar phenomenon observed in a transmembrane TNF-related Fas ligand (FasL) which undergoes metalloproteinase-mediated proteolytic cleavage to release the shed form of the ligand (sFasL). It was found that the apoptotic-inducing capacity of sFasL was reduced by 1,000-fold compared to transmembrane FasL [40]. Similarly, the distinct roles of transmembrane and shed forms of Syndecan-1 (Sdc1) in breast cancer progression were revealed [41]. Overexpression of wild type Sdc1 promoted cell proliferation, whereas its shed form inhibited proliferation. In contrast, the transmembrane Sdc1 inhibited invasiveness whereas soluble Sdc1 vastly promoted the invasion of MCF-7 in vitro [41].

On the basis of the previous comparative studies between transmembrane protein and its extracellular form, we speculated the functional difference between mRANKL and RANKL-Ex. For functional and structural studies, qualitative and quantitative production of proteins are essential. Yet, high level expression of stable and functional mammalian proteins, particularly membrane proteins, remains a challenging task. Moreover, there are several potential bottlenecks such as codon usage, expression systems and/or conditions that might affect both yield and solubility of membrane proteins in *E. coli*. In recent years, the significant divergent codon bias between the *E. coli* and mammalian genes are balanced by engineering the genes to preferentially used synonymous codons compatible with the host [27].

In this study, we explored the use of codon optimization and response surface methodology to achieve the high level expression of mRANKL in *E. coli*. Among different vector systems and *E. coli* hosts, SHuffle Express *E. coli* and pMAL-c5X possessing P*_tac_* promoter were found to be most appropriate host and vector system, respectively for the expression of mRANKL in soluble form. We obtained significant amount of mRANKL from SHuffle *E. coli*-pOmR-c5X when induced with lactose. Among the three different solubility tags, i.e., MBP-, GST-, and His_6_-tag, MBP was found to be the most effective solubilizing agent for the expression of mRANKL in SHuffle Express *E. coli*. MBP is most commonly used as an N-terminal tag for cytosolic expression of the protein. As a fusion tag, it facilitates expression, solubility, and purification [42]. Initially, soluble mRANKL was not obtained from SHuffle *E. coli*-pOmR-c5X when IPTG was used as an inducer. As an alternative, lactose was selected because natural lactose-induction provides several advantages over the IPTG-induction such as low cost, metabolizable and non-toxicity [43].

After the selection of suitable expression system, we aimed to optimize several factors (cell density before induction, lactose concentration, post-induction temperature and post-induction time) for the high production of soluble proteins. Due to several parameters, we explored CCD and RSM to fix and evaluate the interactive effects of most influential parameters of culture condition. The five levels, four factors, CCD at the given range of the above mentioned parameters predicted a set of 30 combination of variables to optimize the expression of mRANKL in *E. coli*. The response (mRANKL (mg/L) was correlated with the four factors as a function of the second-order polynomial equation, given by Eq. 1, indicating that linear coefficients A (OD_600_ before induction), B (lactose concentration), C (post-induction temperature) and D (post-induction time) exhibited a significant impact on production of mRANKL. ANOVA analyses resulted high F-value, very low probability (p > F) value and statistically insignificant lack of fit test at 95% confidence level indicating that the model was adequate for representing the experimental data. The adequacy of quadratic model was confirmed by the high coefficient of determination, R^2^, and the adjusted determination coefficient, R^2^_adj_, value both of which are close to 1, ensuring a high correlation between the experimental values and the predicted values.

Furthermore, in order to gain a better understanding of the four factors for optimal production of mRANKL, the models were presented as 3-D response surfaces. The optimum conditions for mRANKL production was found to be at OD_600_ of 0.6, lactose concentration of 7.5 mM, post-induction temperature of 26°C and post-induction time of 5 h. These plots showed that the production of mRANKL was found to be increased with increasing OD_600_ and post induction time. However, an increase in both factors beyond the optimum region (OD_600_: 0.6 and induction time: 5 h) resulted in a decrease in mRANKL production. While increased production of mRANKL was observed with increasing post-induction temperature, the lactose concentration showed less effect on the production of mRANKL. However, at the temperatures higher than 30°C, the production of mRANKL began to decrease at all OD_600_. While mRANKL as inclusion bodies was produced at high levels above 30°C, high yield of soluble mRANKL was produced when temperature was down-shifted to 26°C. Generally, protein expression processes operate a biphasic culture whereby cells are grown at 37°C to maximise biomass and then the culture is shifted to a lower temperature (25–30°C) while maintaining a longer and more viable stationary/production phase. It is known that the expression of the proteins at low temperatures usually improves both solubility and activity of proteins by increasing stability and correct folding patterns [44]. The results obtained from the experiment were found to be in good agreement with the values predicted by the model. In our laboratory condition, the maximum of 52.4 mg/L of purified MBP-tagged mRANKL was obtained under optimum conditions. Thus, OD_600_ lesser than 0.7, post-induction time of 5 h and lower post-induction temperature values are favorable for the production mRANKL while the concentration of lactose has less influence on the production of mRANKL.

Since we aimed to perform a comparative functional study between mRANKL and extracellular RANKL-Ex, the expression of RANKL-Ex was also optimized in SHuffle Express *E. coli* by CDD and RSM approach. We successfully produced about 130.8 mg/L of purified MBP-tagged RANKL-Ex by optimization of expression conditions whereas Papaneophytou et al., succeeded to produce 11.4 mg/L of extracellular RANKL in *E. coli* host using RSM [45]. Both mRANKL and RANKL-Ex were separated from their fusion partners prior to analysis of their biological activities.

It has been well established that extracellular RANKL self-aggregates into homotrimer and the trimeric form is essential for the activation of its cognate receptor RANK. Therefore, it is necessary to determine the native state of mRANKL prior to test its biological activity. The native form of mRANKL was determined by native gel electrophoresis and gel filtration chromatography. Both experiments showed that mRANKL exist as homotrimer by self-assembly. To distinguish between the roles of transmembrane and extracellular forms of RANKL, the efficiency of mRANKL and RANKL-Ex to induce osteoclastogenesis in RAW264.7 cells was tested and analyzed by TRAP staining and TRAP assay. It was observed that mRANKL is approximately 2-fold more potent than RANKL-Ex in inducing the osteoclastogenesis of RAW264.7 cells. In a similar experiment, the extracellular RANKL was oligomerized to mimic the function of transmembrane RANKL and tested its function in osteoclastogenesis [46]. The experiment demonstrated that oligomerized RANKL works more efficiently than extracellular RANKL in the generation of osteoclasts. Consistently, our results clearly demonstrated that the biological activity of mRANKL is comparatively higher than RANKL-Ex.

Osteoclast differentiation is associated with up-regulation of specific genes in response to RANKL. Osteoclasts express several markers, such as TRAP, CALCR and CFMS throughout the differentiation process which, along with multinucleation and resorption, characterize the osteoclast phenotype [47]. CALCR is expressed on the surface of mature osteoclast and it has been described as the best differentiation marker for the osteoclast [48]. CFMS triggers the proliferation and fusion of mononuclear cells to form multinucleated, mature osteoclasts [49]. NFATc1 is believed to be a master transcription factor for murine osteoclastogenesis [50]. RANKL signaling cascade plays a significant role in the regulation of cathepsin K expression. In fact RANKL stimulates the osteoclast to produce increased amounts of cathepsin K [51]. Our qRT-PCR analyses revealed that mRANKL significantly upregulates the genes that are induced during osteoclast differentiation. Not only the assay of TRAP activity showed that mRANKL is more efficient to induce osteoclastogenesis *in vitro* compared to RANKL-Ex, but quantitative RT-PCR assays also showed that mRANKL is approximately twice as much active as RANKL-Ex.

In conclusion, we successfully demonstrated the high level expression of both mRANKL and RANKL-Ex in soluble and active forms by codon optimization and response surface methodology. It demonstrates that the codon optimized synthetic gene products expressed in *E. coli* retain their functional properties. Therefore, it will offer great opportunity for structural and functional studies on transmembrane proteins. Importantly, our study also revealed that mRANKL is more potent than extracellular RANKL to induce osteoclastogenesis. We hope these findings will certainly contribute to broaden the insight of diverse roles of mRANKL, and to understand the RANKL/RANK system.

## Supporting Information

Figure S1 Diagnostic plots for estimating the adequacy of the regression model. Correlation between predicted and actual value for RANKL-Ex production (A). The studentized and normal percentage probability plot of RANKL-Ex production (B).
(TIF)

Figure S2 Response surface plots to visualize the relationship between the response and experimental levels of each factor. Three dimensional surface plots of combined effect of cell density (OD_600_) and lactose concentration (A), OD_600_ and post-induction temperature (B), OD_600_ and post-induction time (C), post-induction temperature and lactose concentration (D), post-induction time and lactose concentration (E) and post- induction temperature and post-induction time (F) on RANKL-Ex production.
(TIF)

Figure S3 SDS PAGE and Western blot analyses of RANKL-Ex fusion proteins. A: Comassie blue stained SDS gel showing lysates from two SHuffle *E. coli*-pOsREx-c5x colonies expressing the RANKL-Ex fusion proteins (∼62 kDa). Lane 1: Protein standards, molecular masses are indicated in kilodaltons (kDa); Lanes 2 and 3: Insoluble and soluble fractions from clone 1, respectively; Lanes 4 and 5: Insoluble and soluble fractions from clone 2, respectively. B: Comassie blue stained SDS gel showing purification of crude RANKL-Ex using amylose resin. Lane 1: crude soluble RANKL-Ex; Lane 2: Flow through; Lane 3: Wash flow through; Lane 4: Protein standards, molecular masses are indicated in kilodaltons (kDa); Lanes 5–7: Elution fractions. C: Western blot analysis of purified RANKL-Ex. Different amount of purified RANKL-Ex was run in 4–20% SDS page and transferred onto a nitrocellulose membrane. Detection was performed with anti-RANKL primary antibody, goat IgG HRP-conjugated secondary antibody and chemiluminescent substrate. Lane 1: Protein marker; Lanes 2–5: 200, 100, 50 and 30 ng of purified RANKL-Ex protein respectively.
(TIF)

Table S1 Codon usage table.
(DOCX)

Table S2 CCD with measured and predicted response of RANKL-Ex production.
(DOCX)

Table S3 ANOVA for response surface quadratic model for RANKL-Ex production.
(DOCX)

Table S4 Confirmation experiments for RANKL-Ex production.
(DOCX)
