# Correction: Exploring Codon Optimization and Response Surface Methodology to Express Biologically Active Transmembrane RANKL in *E. coli*

**DOI:** 10.1371/journal.pone.0107314

**Published:** 2014-09-12

**Authors:** The PLOS ONE Staff

The following information is missing from the Funding section: This research is supported by Animal Disease Management Technology Development, Ministry of Agriculture, Food and Rural Affairs, Republic of Korea.
