# Supplementary figures and images for: Exploring Codon Optimization and Response Surface Methodology to Express Biologically Active Transmembrane RANKL in E. coli
Source: PLoS One. 2014 May 8;9(5):e96259. doi: 10.1371/journal.pone.0096259 (PMC4014495; doi:10.1371/journal.pone.0096259)

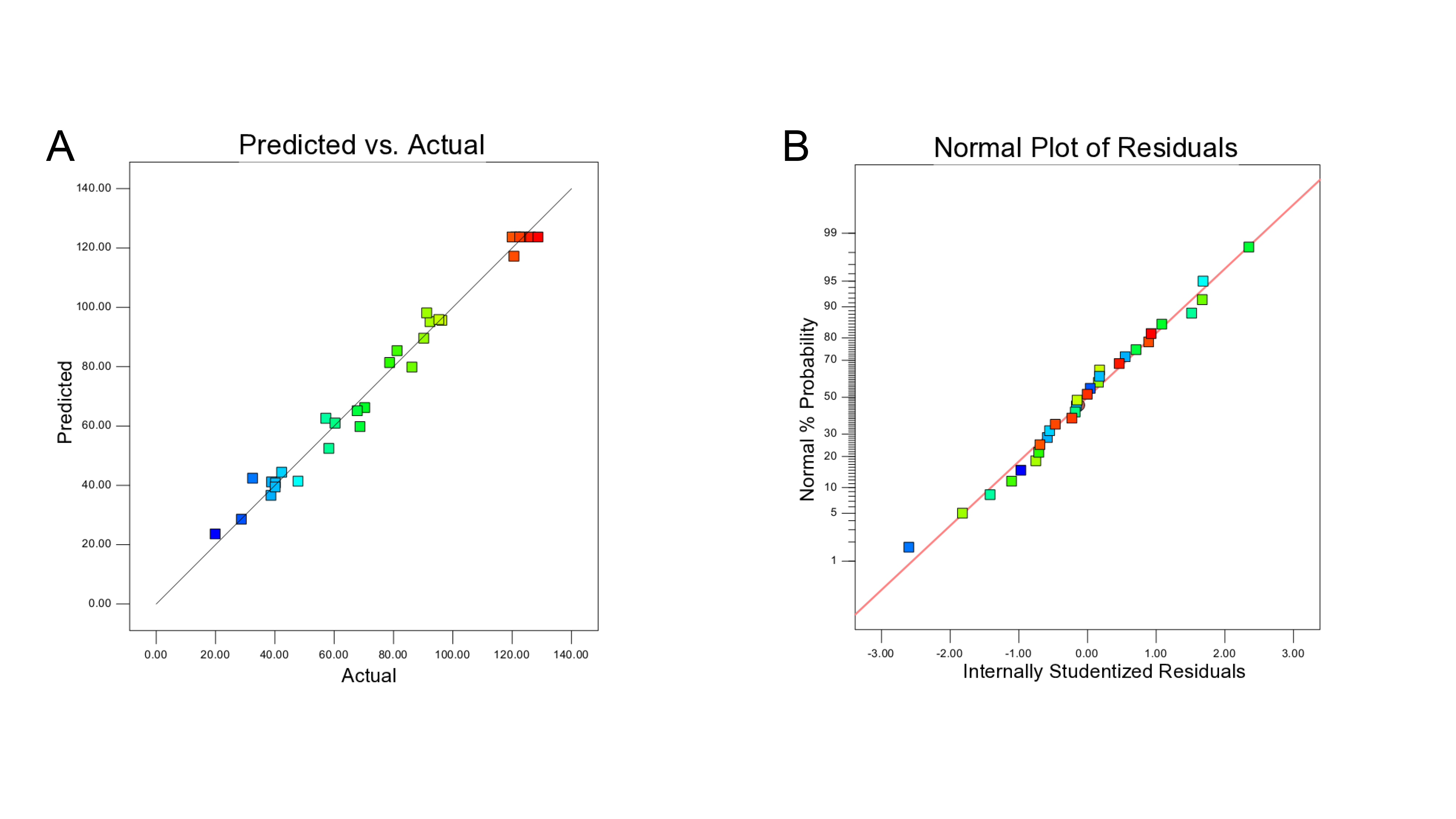

Supplement: Figure S1 — Diagnostic plots for estimating the adequacy of the regression model. Correlation between predicted and actual value for RANKL-Ex production (A). The studentized and normal percentage probability plot of RANKL-Ex production (B). (TIF) [file pone.0096259.s001.tif]

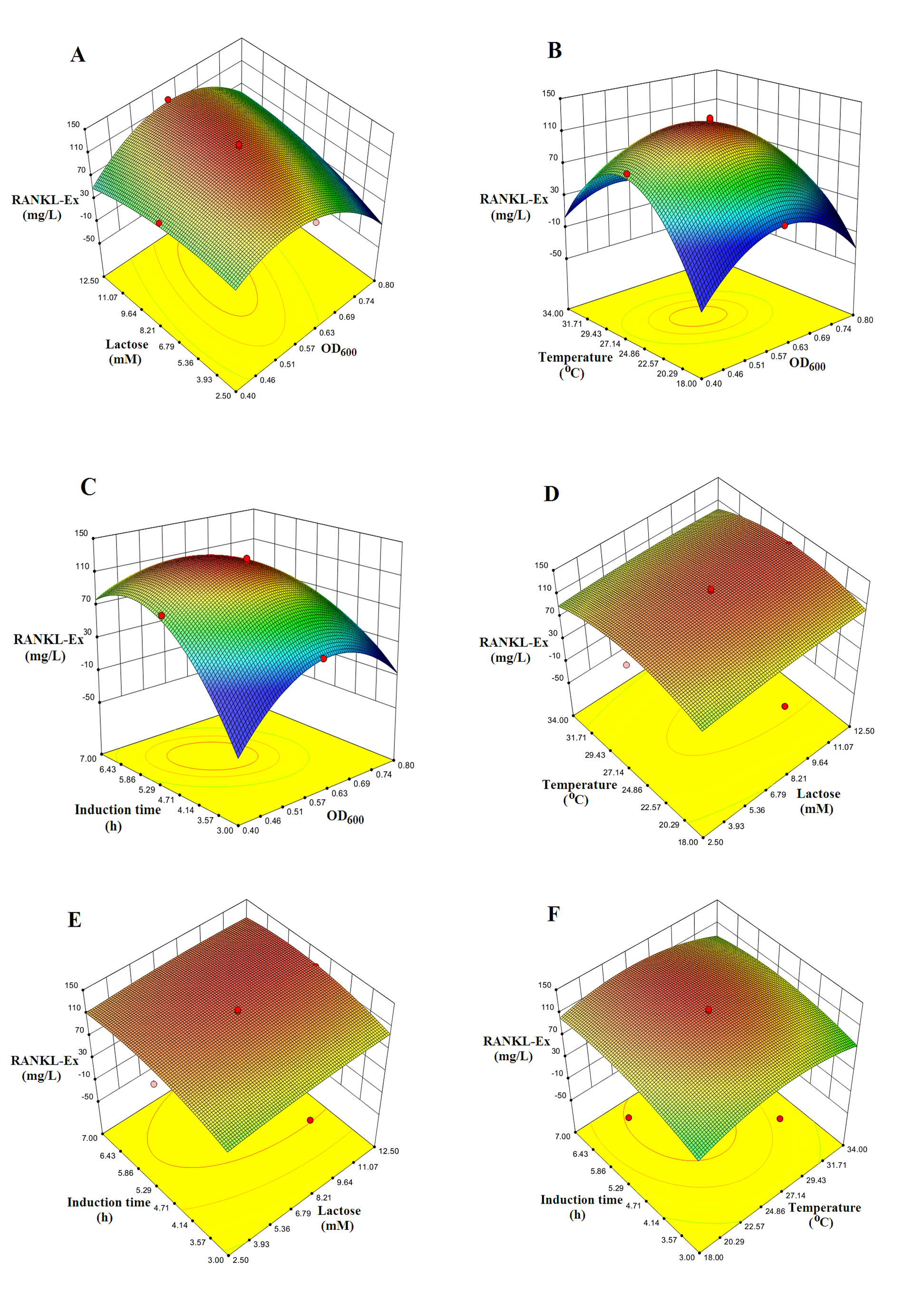

Supplement: Figure S2 — Response surface plots to visualize the relationship between the response and experimental levels of each factor. Three dimensional surface plots of combined effect of cell density (OD600) and lactose concentration (A), OD600 and post-induction temperature (B), OD600 and post-induction time (C), post-induction temperature and lactose concentration (D), post-induction time and lactose concentration (E) and post- induction temperature and post-induction time (F) on RANKL-Ex production. (TIF) [file pone.0096259.s002.tif]

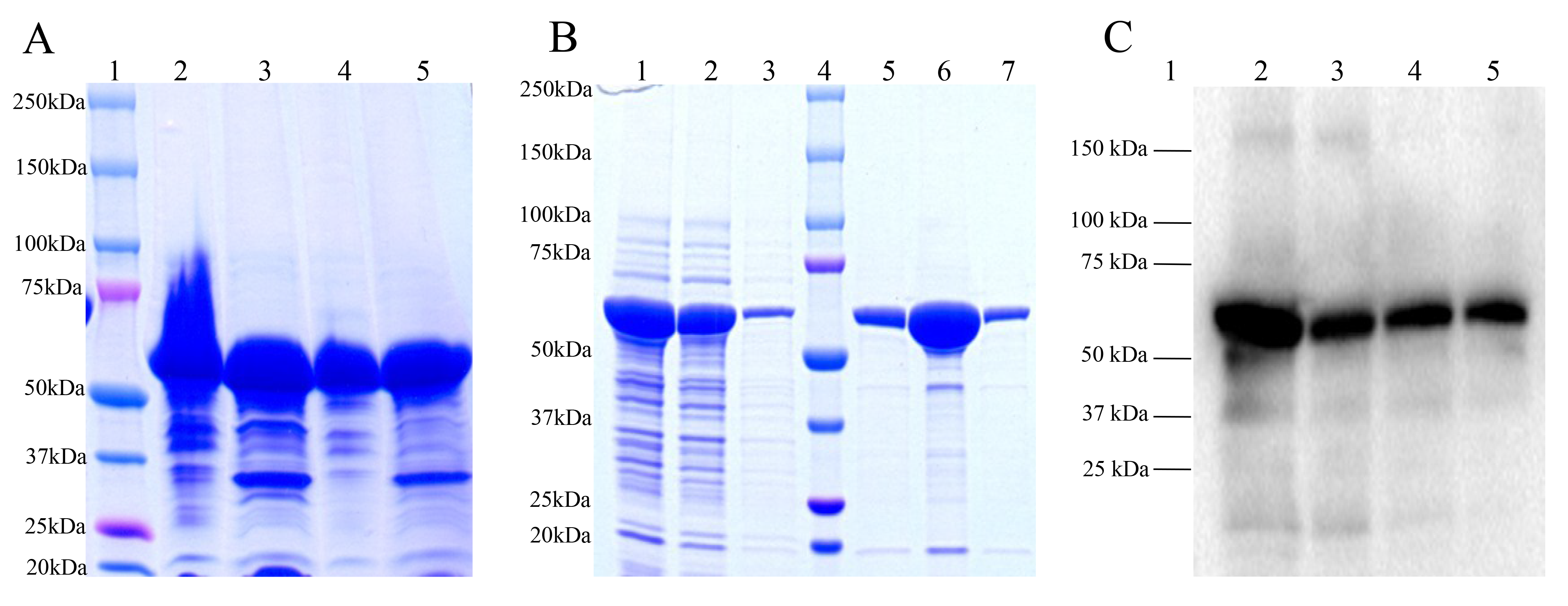

Supplement: Figure S3 — SDS PAGE and Western blot analyses of RANKL-Ex fusion proteins. A: Comassie blue stained SDS gel showing lysates from two SHuffle E. coli-pOsREx-c5x colonies expressing the RANKL-Ex fusion proteins (∼62 kDa). Lane 1: Protein standards, molecular masses are indicated in kilodaltons (kDa); Lanes 2 and 3: Insoluble and soluble fractions from clone 1, respectively; Lanes 4 and 5: Insoluble and soluble fractions from clone 2, respectively. B: Comassie blue stained SDS gel showing purification of crude RANKL-Ex using amylose resin. Lane 1: crude soluble RANKL-Ex; Lane 2: Flow through; Lane 3: Wash flow through; Lane 4: Protein standards, molecular masses are indicated in kilodaltons (kDa); Lanes 5–7: Elution fractions. C: Western blot analysis of purified RANKL-Ex. Different amount of purified RANKL-Ex was run in 4–20% SDS page and transferred onto a nitrocellulose membrane. Detection was performed with anti-RANKL primary antibody, goat IgG HRP-conjugated secondary antibody and chemiluminescent substrate. Lane 1: Protein marker; Lanes 2–5: 200, 100, 50 and 30 ng of purified RANKL-Ex protein respectively. (TIF) [file pone.0096259.s003.tif]
