# Supplementary material for: Exploring Codon Optimization and Response Surface Methodology to Express Biologically Active Transmembrane RANKL in E. coli
Source: PLoS One. 2014 May 8;9(5):e96259. doi: 10.1371/journal.pone.0096259 (PMC4014495; doi:10.1371/journal.pone.0096259)
Supplement: Table S1 — Codon usage table. (DOCX) [file pone.0096259.s004.docx]

Table S1.

|  |  | RANKL | | | mRANKL | | |  |  | RANKL | | | mRANKL | | |
| --- | --- | --- | --- | --- | --- | --- | --- | --- | --- | --- | --- | --- | --- | --- | --- |
| AA | Codon | Number | /1000 | Fraction | Number | /1000 | Fraction | AA | Codon | Number | /1000 | Fraction | Number | /1000 | Fraction |
| Gly | GGG | 6 | 18.93 | 0.23 | 0 | 0 | 0 | Trp | TGG | 4 | 12.62 | 1 | 4 | 12.66 | 1 |
| Gly | GGA | 6 | 18.93 | 0.23 | 0 | 0 | 0 | End | TGA | 1 | 3.15 | 1 | 0 | 0 | 0 |
| Gly | GGT | 4 | 12.62 | 0.15 | 22 | 69.62 | 0.85 | Cys | TGT | 0 | 0 | 0 | 0 | 0 | 0 |
| Gly | GGC | 10 | 31.55 | 0.38 | 4 | 12.66 | 0.15 | Cys | TGC | 4 | 12.62 | 1 | 4 | 12.66 | 1 |
|  |  |  |  |  |  |  |  |  |  |  |  |  |  |  |  |
| Glu | GAG | 3 | 9.46 | 0.21 | 4 | 12.66 | 0.29 | End | TAG | 0 | 0 | 0 | 0 | 0 | 0 |
| Glu | GAA | 11 | 34.7 | 0.79 | 10 | 31.65 | 0.71 | End | TAA | 0 | 0 | 0 | 0 | 0 | 0 |
| Asp | GAT | 7 | 22.08 | 0.47 | 5 | 15.82 | 0.33 | Tyr | TAT | 5 | 15.77 | 0.42 | 3 | 9.49 | 0.25 |
| Asp | GAC | 8 | 25.24 | 0.53 | 10 | 31.65 | 0.67 | Tyr | TAC | 7 | 22.08 | 0.58 | 9 | 28.48 | 0.75 |
|  |  |  |  |  |  |  |  |  |  |  |  |  |  |  |  |
| Val | GTG | 6 | 18.93 | 0.4 | 3 | 9.49 | 0.2 | Leu | TTG | 2 | 6.31 | 0.07 | 0 | 0 | 0 |
| Val | GTA | 1 | 3.15 | 0.07 | 1 | 3.16 | 0.07 | Leu | TTA | 1 | 3.15 | 0.04 | 0 | 0 | 0 |
| Val | GTT | 4 | 12.62 | 0.27 | 10 | 31.65 | 0.67 | Phe | TTT | 8 | 25.24 | 0.53 | 3 | 9.49 | 0.2 |
| Val | GTC | 4 | 12.62 | 0.27 | 1 | 3.16 | 0.07 | Phe | TTC | 7 | 22.08 | 0.47 | 12 | 37.97 | 0.8 |
|  |  |  |  |  |  |  |  |  |  |  |  |  |  |  |  |
| Ala | GCG | 4 | 12.62 | 0.16 | 17 | 53.8 | 0.68 | Ser | TCG | 5 | 15.77 | 0.15 | 0 | 0 | 0 |
| Ala | GCA | 3 | 9.46 | 0.12 | 1 | 3.16 | 0.04 | Ser | TCA | 3 | 9.46 | 0.09 | 0 | 0 | 0 |
| Ala | GCT | 7 | 22.08 | 0.28 | 4 | 12.66 | 0.16 | Ser | TCT | 5 | 15.77 | 0.15 | 22 | 69.62 | 0.65 |
| Ala | GCC | 11 | 34.7 | 0.44 | 3 | 9.49 | 0.12 | Ser | TCC | 8 | 25.24 | 0.24 | 6 | 18.99 | 0.18 |
|  |  |  |  |  |  |  |  |  |  |  |  |  |  |  |  |
| Arg | AGG | 3 | 9.46 | 0.18 | 0 | 0 | 0 | Arg | CGG | 2 | 6.31 | 0.12 | 0 | 0 | 0 |
| Arg | AGA | 3 | 9.46 | 0.18 | 0 | 0 | 0 | Arg | CGA | 5 | 15.77 | 0.29 | 0 | 0 | 0 |
| Ser | AGT | 2 | 6.31 | 0.06 | 0 | 0 | 0 | Arg | CGT | 0 | 0 | 0 | 14 | 44.3 | 0.82 |
| Ser | AGC | 11 | 34.7 | 0.32 | 6 | 18.99 | 0.18 | Arg | CGC | 4 | 12.62 | 0.24 | 3 | 9.49 | 0.18 |
|  |  |  |  |  |  |  |  |  |  |  |  |  |  |  |  |
| Lys | AAG | 5 | 15.77 | 0.38 | 3 | 9.49 | 0.23 | Gln | CAG | 11 | 34.7 | 0.73 | 10 | 31.65 | 0.67 |
| Lys | AAA | 8 | 25.24 | 0.62 | 10 | 31.65 | 0.77 | Gln | CAA | 4 | 12.62 | 0.27 | 5 | 15.82 | 0.33 |
| Asn | AAT | 3 | 9.46 | 0.25 | 2 | 6.33 | 0.17 | His | CAT | 5 | 15.77 | 0.42 | 5 | 15.82 | 0.42 |
| Asn | AAC | 9 | 28.39 | 0.75 | 10 | 31.65 | 0.83 | His | CAC | 7 | 22.08 | 0.58 | 7 | 22.15 | 0.58 |
|  |  |  |  |  |  |  |  |  |  |  |  |  |  |  |  |
| Met | ATG | 10 | 31.55 | 1 | 10 | 31.65 | 1 | Leu | CTG | 17 | 53.63 | 0.63 | 23 | 72.78 | 0.85 |
| Ile | ATA | 3 | 9.46 | 0.21 | 0 | 0 | 0 | Leu | CTA | 2 | 6.31 | 0.07 | 0 | 0 | 0 |
| Ile | ATT | 4 | 12.62 | 0.29 | 5 | 15.82 | 0.36 | Leu | CTT | 1 | 3.15 | 0.04 | 0 | 0 | 0 |
| Ile | ATC | 7 | 22.08 | 0.5 | 9 | 28.48 | 0.64 | Leu | CTC | 4 | 12.62 | 0.15 | 4 | 12.66 | 0.15 |
|  |  |  |  |  |  |  |  |  |  |  |  |  |  |  |  |
| Thr | ACG | 3 | 9.46 | 0.27 | 3 | 9.49 | 0.27 | Pro | CCG | 5 | 15.77 | 0.24 | 14 | 44.3 | 0.67 |
| Thr | ACA | 3 | 9.46 | 0.27 | 0 | 0 | 0 | Pro | CCA | 7 | 22.08 | 0.33 | 4 | 12.66 | 0.19 |
| Thr | ACT | 3 | 9.46 | 0.27 | 1 | 3.16 | 0.09 | Pro | CCT | 6 | 18.93 | 0.29 | 3 | 9.49 | 0.14 |
| Thr | ACC | 2 | 6.31 | 0.18 | 7 | 22.15 | 0.64 | Pro | CCC | 3 | 9.46 | 0.14 | 0 | 0 | 0 |
