# Supplementary material for: Exploring Codon Optimization and Response Surface Methodology to Express Biologically Active Transmembrane RANKL in E. coli
Source: PLoS One. 2014 May 8;9(5):e96259. doi: 10.1371/journal.pone.0096259 (PMC4014495; doi:10.1371/journal.pone.0096259)
Supplement: Table S2 — CCD with measured and predicted response of RANKL-Ex production. (DOCX) [file pone.0096259.s005.docx]

Table S2.

| Run | A  OD600 | B  Lactose (mM) | C  Temperature (℃) | D  Induction time (h) | Response  RANKL-Ex (mg/L) | |
| --- | --- | --- | --- | --- | --- | --- |
|  |  |  |  |  | Predicted value | Actual value |
| 1 | -1 | -1 | -1 | -1 | 41.1 | 38.9 |
| 2 | 1 | -1 | -1 | -1 | 40.8 | 40.2 |
| 3 | -1 | 1 | -1 | -1 | 44.3 | 42.3 |
| 4 | 1 | 1 | -1 | -1 | 66.1 | 70.3 |
| 5 | -1 | -1 | 1 | -1 | 65.1 | 67.8 |
| 6 | 1 | -1 | 1 | -1 | 41.4 | 47.8 |
| 7 | -1 | 1 | 1 | -1 | 62.5 | 57.2 |
| 8 | 1 | 1 | 1 | -1 | 60.9 | 60.3 |
| 9 | -1 | -1 | -1 | 1 | 95.6 | 96.3 |
| 10 | 1 | -1 | -1 | 1 | 59.7 | 68.7 |
| 11 | -1 | 1 | -1 | 1 | 95.1 | 92.3 |
| 12 | 1 | 1 | -1 | 1 | 81.3 | 78.7 |
| 13 | -1 | -1 | 1 | 1 | 95.8 | 95.3 |
| 14 | 1 | -1 | 1 | 1 | 36.6 | 38.7 |
| 15 | -1 | 1 | 1 | 1 | 89.5 | 90.2 |
| 16 | 1 | 1 | 1 | 1 | 52.4 | 58.2 |
| 17 | -2 | 0 | 0 | 0 | 79.8 | 86.2 |
| 18 | 2 | 0 | 0 | 0 | 42.3 | 32.5 |
| 19 | 0 | -2 | 0 | 0 | 98.1 | 91.2 |
| 20 | 0 | 2 | 0 | 0 | 117.2 | 120.6 |
| 21 | 0 | 0 | -2 | 0 | 28.5 | 28.7 |
| 22 | 0 | 0 | 2 | 0 | 23.5 | 19.9 |
| 23 | 0 | 0 | 0 | -2 | 39.4 | 40.1 |
| 24 | 0 | 0 | 0 | 2 | 85.4 | 81.2 |
| 25 | 0 | 0 | 0 | 0 | 123.7 | 123.7 |
| 26 | 0 | 0 | 0 | 0 | 123.7 | 126.2 |
| 27 | 0 | 0 | 0 | 0 | 123.7 | 121.2 |
| 28 | 0 | 0 | 0 | 0 | 123.7 | 120 |
| 29 | 0 | 0 | 0 | 0 | 123.7 | 122.5 |
| 30 | 0 | 0 | 0 | 0 | 123.7 | 128.7 |
