# Supplementary material for: Exploring Codon Optimization and Response Surface Methodology to Express Biologically Active Transmembrane RANKL in E. coli
Source: PLoS One. 2014 May 8;9(5):e96259. doi: 10.1371/journal.pone.0096259 (PMC4014495; doi:10.1371/journal.pone.0096259)
Supplement: Table S3 — ANOVA for response surface quadratic model for RANKL-Ex production. (DOCX) [file pone.0096259.s006.docx]

Table S3.

| **Source** | **df** | **Sum of Squares** | **Mean Square** | **F- Value** | **p-value**  **Prob > F** |
| --- | --- | --- | --- | --- | --- |
| Model | 14 | 32419.181 | 2315.6558 | 66.840287 | < 0.0001 |
| A-OD600 | 1 | 2105.6267 | 2105.6267 | 60.777899 | < 0.0001 |
| B-Lactose | 1 | 547.215 | 547.215 | 15.795097 | 0.0012 |
| C-Temperature | 1 | 37.001667 | 37.001667 | 1.0680353 | 0.3178 |
| D-Induction time | 1 | 3169.4017 | 3169.4017 | 91.483251 | < 0.0001 |
| AB | 1 | 488.41 | 488.41 | 14.097719 | 0.0019 |
| AC | 1 | 547.56 | 547.56 | 15.805055 | 0.0012 |
| AD | 1 | 1263.8025 | 1263.8025 | 36.47905 | < 0.0001 |
| BC | 1 | 33.64 | 33.64 | 0.9710024 | 0.3401 |
| BD | 1 | 14.0625 | 14.0625 | 0.4059073 | 0.5337 |
| CD | 1 | 564.0625 | 564.0625 | 16.281392 | 0.0011 |
| A^2 | 1 | 6717.8743 | 6717.8743 | 193.9082 | < 0.0001 |
| B^2 | 1 | 441.60429 | 441.60429 | 12.746695 | 0.0028 |
| C^2 | 1 | 16346.61 | 16346.61 | 471.83702 | < 0.0001 |
| D^2 | 1 | 6441.7543 | 6441.7543 | 185.93813 | < 0.0001 |
| Residual | 15 | 519.66917 | 34.644611 |  |  |
| Lack of Fit | 10 | 467.04083 | 46.704083 | 4.4371615 | 0.0569 |
| Pure Error | 5 | 52.628333 | 10.525667 |  |  |

A=OD_600_; B=Lactose concentration; C=Temperature; D=Induction time; df = Degrees of freedom
