# Supplementary material for: Exploring Codon Optimization and Response Surface Methodology to Express Biologically Active Transmembrane RANKL in E. coli
Source: PLoS One. 2014 May 8;9(5):e96259. doi: 10.1371/journal.pone.0096259 (PMC4014495; doi:10.1371/journal.pone.0096259)
Supplement: Table S4 — Confirmation experiments for RANKL-Ex production. (DOCX) [file pone.0096259.s007.docx]

Table S4.

|  | Expression condition | | | | RANKL-Ex  (mg/L) |
| --- | --- | --- | --- | --- | --- |
|  | OD_600_ | Lactose (mM) | Temperature  (^o^C) | Induction time (h) |  |
| Predicted value | 0.6 | 7.5 | 26 | 5 | 128.7 |
| Experimental value | 0.6 | 7.5 | 26 | 5 | 130.8 |
